# Supplementary material for: Advancing apple genetics research: Malus coronaria and Malus ioensis genomes and a gene family-based pangenome of native North American apples
Source: DNA Res. 2024 Aug 26;31(5):dsae026. doi: 10.1093/dnares/dsae026 (PMC11489038; doi:10.1093/dnares/dsae026)
Supplement: dsae026_suppl_Supplementary_Material [file dsae026_suppl_supplementary_material.docx]

**Supplementary Files**

**Advancing apple genetics research: *Malus* *coronaria* and *Malus* *ioensis* genomes and a gene family-based pangenome of native North American apples**

Anže Švara^1^, Honghe Sun^2,3^, Zhangjun Fei^2,4^, Awais Khan^1,^*

^1^Plant Pathology and Plant-Microbe Biology Section, School of Integrative Plant Science, Cornell University, Geneva, NY 14456, USA

^2^Boyce Thompson Institute, Cornell University, Ithaca, NY 14853, USA

^3^Plant Biology Section, School of Integrative Plant Science, Cornell University, Ithaca, NY 14853, USA

^4^USDA-ARS Robert W. Holley Center for Agriculture and Health, Ithaca, NY 14853, USA

*Corresponding author ([awais.khan@cornell.edu](mailto:awais.khan@cornell.edu))

**Supplementary Figures**


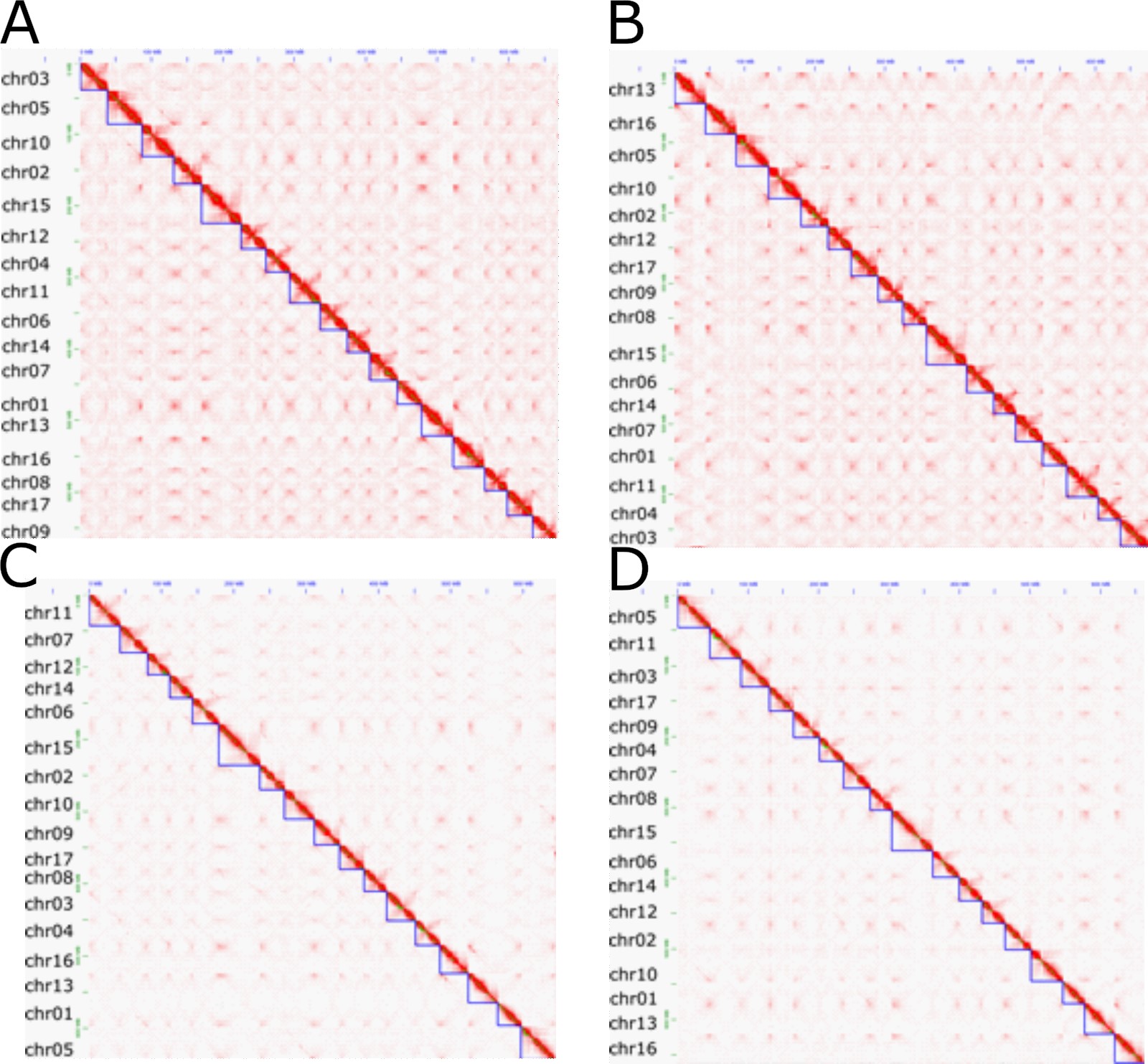


**Figure S1:** Chromatin contact maps of *Malus coronaria* accession PI590014 A) haplome 1 and B) 2, and *Malus ioensis* accession PI590015 haplome C) 1 and D) 2.


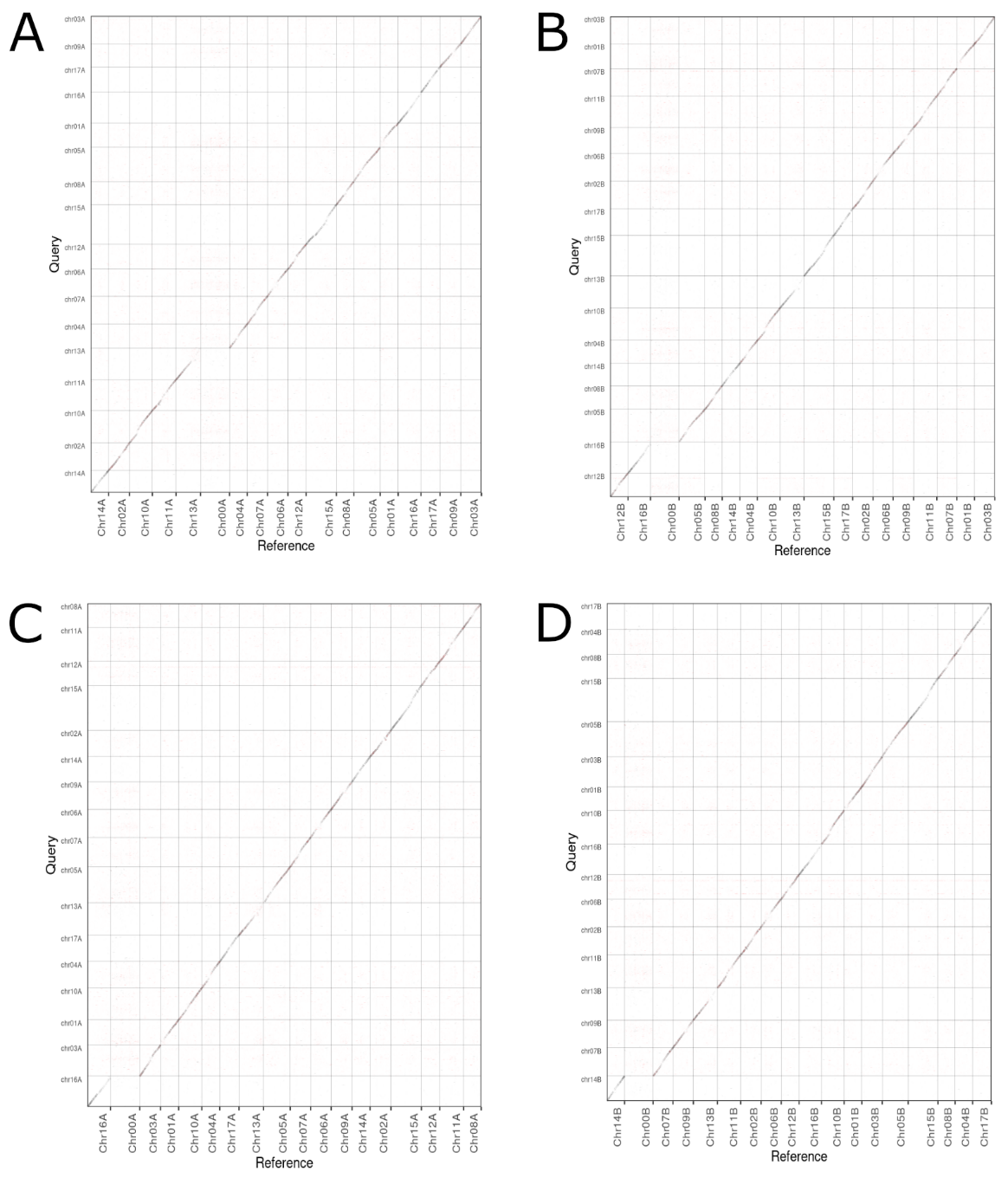


**Figure S2:** Collinearity between *Malus coronaria* accession PI590014 haplomes A) 1 and B) 2 and C) haplome 1 and D) 2 of *Malus ioensis* accession PI590015 with GDDH13 genome [31].


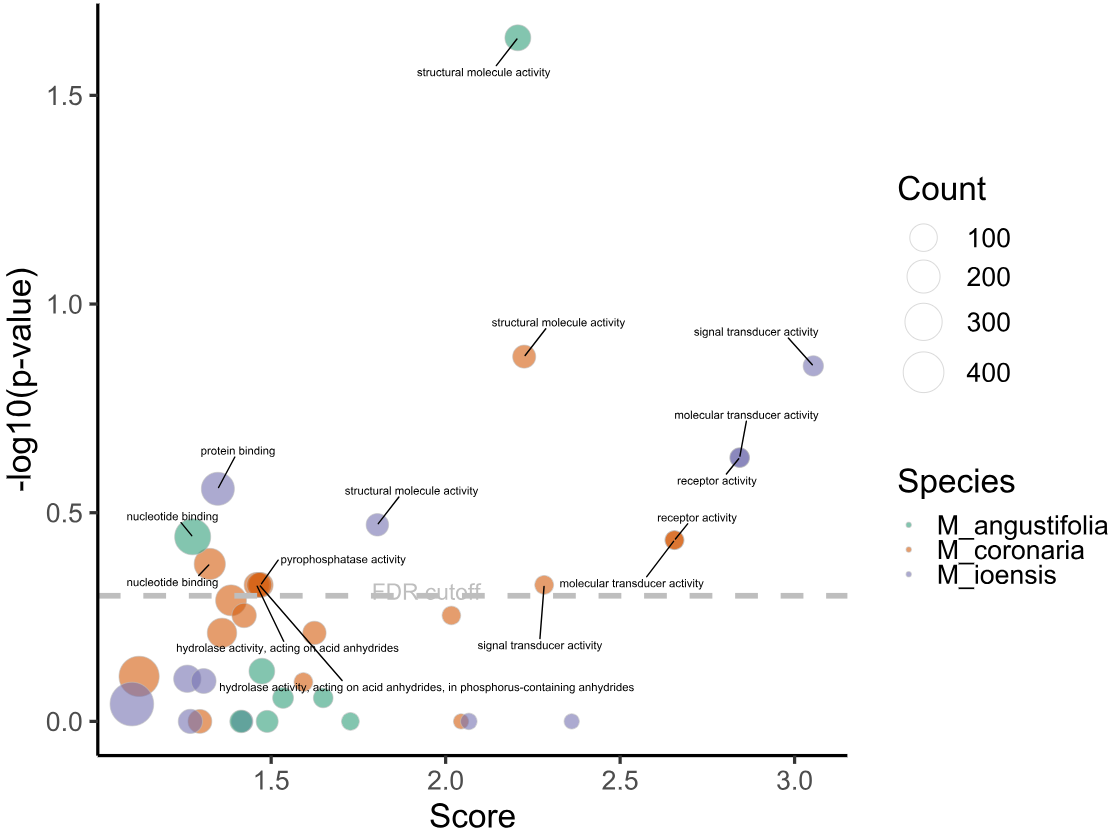


**Fig S3:** Enrichment of processes associated with genes of the orthogroups belonging to species-specific genomes of *Malus coronaria*, *M. ioensis*, and of previously-assembled *M. angustifolia*, and *M. fusca* (no significant enrichment found) genomes [3,5].


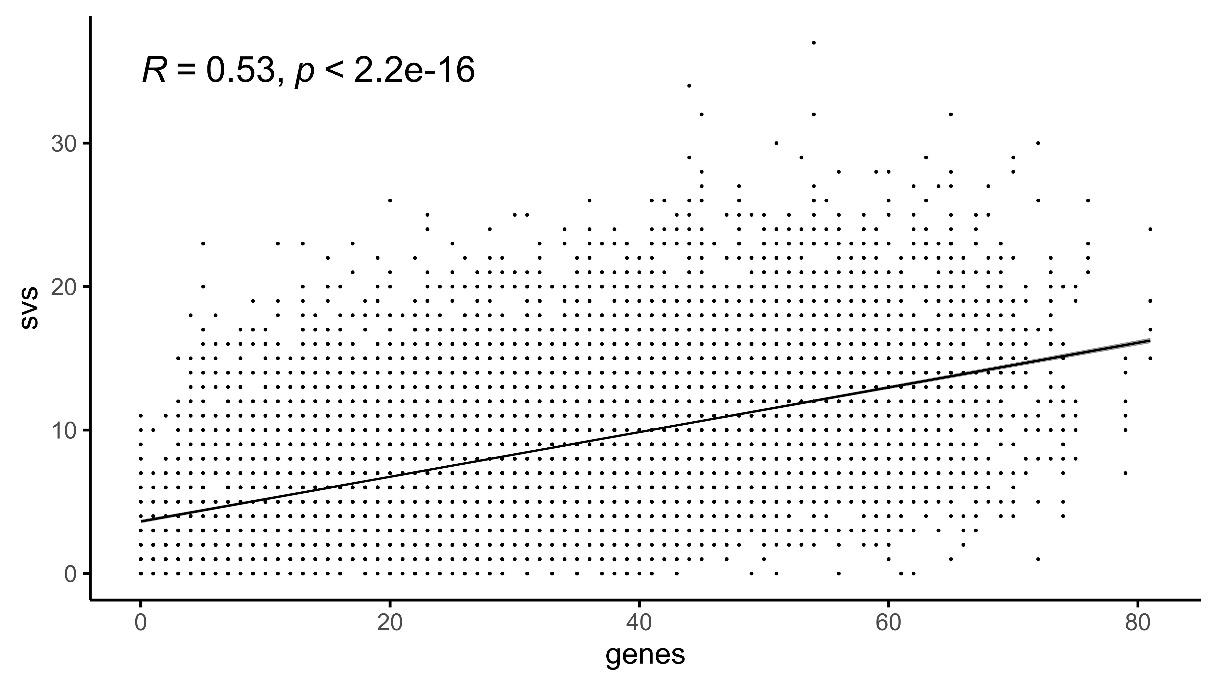


**Fig S4:** Correlation plot showing the number of genes and structural variations within 400 kb regions along the *Malus coronaria*, *M. ioensis*, and of previously-assembled *M. fusca* genome [5].

**Supplementary Tables**

**Table S1**: Repeats summary of *Malus coronaria* (PI590014) and *M. ioensis* (PI590015) haplomes, and of previously-assembled *M. angustifolia* and *M. fusca* haplomes [3,5].

| **8** | | **coronaria haploid 1** | | | **coronaria haploid 2** | | | **ioensis haploid 1** | | | **ioensis haploid 2** | | | **Malus angustifolia haploid 1** | | | **Malus angustifolia haploid 2** | | | **Malus fusca haploid 1** | | | **Malus fusca haploid 2** | | |
| --- | --- | --- | --- | --- | --- | --- | --- | --- | --- | --- | --- | --- | --- | --- | --- | --- | --- | --- | --- | --- | --- | --- | --- | --- | --- |
|  |  | **Count** | **Base pairs** | **%** | **Count** | **Base pairs** | **%** | **Count** | **Base pairs** | **%** | **Count** | **Base pairs** | **%** | **Count** | **Base pairs** | **%** | **Count** | **Base pairs** | **%** | **Count** | **Base pairs** | **%** | **Count** | **Base pairs** | **%** |
| **Class II - DNA transposons** | **CMC-EnSpm** | 19,334 | 6,255,581 | 0.94 | 19,122 | 6,201,449 | 0.92 | 18,744 | 6,078,446 | 0.94 | 19,307 | 6,215,995 | 0.94 | 18,735 | 5,606,391 | 1.19 | 18,485 | 5,555,685 | 1.19 | 18,583 | 5,912,907 | 0.93 | 18,510 | 5,855,058 | 0.93 |
|  | **Dada** | 56 | 6,521 | 0.00 | 57 | 6,565 | 0.00 | 62 | 7,528 | 0.00 | 57 | 6,490 | 0.00 | 59 | 6,369 | 0.00 | 57 | 6,039 | 0.00 | 55 | 6,091 | 0.00 | 59 | 6,378 | 0.00 |
|  | **Ginger** | 115 | 29,279 | 0.00 | 111 | 28,751 | 0.00 | 114 | 29,404 | 0.00 | 112 | 29,708 | 0.00 | 121 | 30,719 | 0.01 | 124 | 31,423 | 0.01 | 116 | 31,185 | 0.00 | 125 | 33,886 | 0.01 |
|  | **MULE-MuDR** | 78,416 | 15,951,954 | 2.39 | 78,688 | 16,071,241 | 2.38 | 77,634 | 15,777,264 | 2.45 | 78,700 | 16,044,012 | 2.43 | 80,263 | 16,158,416 | 3.44 | 80,164 | 16,129,811 | 3.44 | 83,434 | 16,099,991 | 2.53 | 83,111 | 16,071,627 | 2.54 |
|  | **Maverick** | 99 | 12,116 | 0.00 | 93 | 11,714 | 0.00 | 95 | 11,600 | 0.00 | 103 | 13,173 | 0.00 | 101 | 12,326 | 0.00 | 108 | 13,436 | 0.00 | 104 | 14,408 | 0.00 | 105 | 15,027 | 0.00 |
|  | **PIF-Harbinger** | 46,105 | 14,304,521 | 2.15 | 45,849 | 14,212,774 | 2.11 | 45,411 | 14,026,827 | 2.18 | 46,007 | 14,266,506 | 2.16 | 42,018 | 10,695,522 | 2.27 | 42,089 | 10,682,408 | 2.28 | 44,651 | 13,330,403 | 2.09 | 44,894 | 13,378,673 | 2.12 |
|  | **TcMar-Mariner** | 31 | 6,833 | 0.00 | 37 | 5,968 | 0.00 | 39 | 5,943 | 0.00 | 37 | 6,022 | 0.00 | 37 | 5,934 | 0.00 | 34 | 5,875 | 0.00 | 51 | 9,689 | 0.00 | 47 | 8,690 | 0.00 |
|  | **TcMar-Pogo** | 1,051 | 152,798 | 0.02 | 1,055 | 151,533 | 0.02 | 1,010 | 150,272 | 0.02 | 1,064 | 153,893 | 0.02 | 1,107 | 158,514 | 0.03 | 1,063 | 154,383 | 0.03 | 1,017 | 148,344 | 0.02 | 1,015 | 148,737 | 0.02 |
|  | **TcMar-Stowaway** | 78 | 48,968 | 0.01 | 80 | 49,192 | 0.01 | 74 | 47,736 | 0.01 | 52 | 30,881 | 0.00 | 89 | 48,710 | 0.01 | 82 | 48,442 | 0.01 | 83 | 52,813 | 0.01 | 97 | 56,229 | 0.01 |
|  | **hAT-Ac** | 42,466 | 11,912,491 | 1.79 | 42,029 | 11,775,935 | 1.74 | 41,963 | 11,754,345 | 1.83 | 42,425 | 11,910,738 | 1.80 | 31,723 | 7,797,522 | 1.66 | 32,151 | 7,879,398 | 1.68 | 40,861 | 11,597,331 | 1.82 | 40,485 | 11,461,333 | 1.81 |
|  | **hAT-Charlie** | 2,046 | 409,963 | 0.06 | 2,075 | 411,934 | 0.06 | 2,026 | 411,993 | 0.06 | 2,060 | 411,631 | 0.06 | 2,067 | 405,582 | 0.09 | 2,024 | 399,350 | 0.09 | 1,921 | 374,413 | 0.06 | 1,880 | 367,818 | 0.06 |
|  | **hAT-Tag1** | 20,648 | 5,824,459 | 0.87 | 21,026 | 5,942,100 | 0.88 | 20,788 | 5,884,822 | 0.91 | 21,176 | 5,978,436 | 0.90 | 19,818 | 4,559,140 | 0.97 | 19,740 | 4,550,785 | 0.97 | 21,364 | 6,083,372 | 0.95 | 21,302 | 5,975,030 | 0.95 |
|  | **hAT-Tip100** | 23,669 | 5,395,884 | 0.81 | 23,616 | 5,354,202 | 0.79 | 23,307 | 5,353,687 | 0.83 | 23,699 | 5,353,900 | 0.81 | 23,113 | 4,543,921 | 0.97 | 22,921 | 4,487,802 | 0.96 | 25,044 | 5,886,287 | 0.92 | 25,206 | 5,900,670 | 0.93 |
|  | **Helitron** | 16,963 | 5,294,616 | 0.79 | 16,803 | 5,212,219 | 0.77 | 16,536 | 5,115,503 | 0.79 | 16,909 | 5,263,986 | 0.80 | 16,578 | 5,206,049 | 1.11 | 16,503 | 5,091,776 | 1.09 | 16,846 | 4,963,323 | 0.78 | 16,618 | 4,835,066 | 0.77 |
|  | **Unknown** | 55,481 | 10,131,085 | 1.52 | 55,615 | 10,173,571 | 1.51 | 54,987 | 10,066,445 | 1.56 | 55,680 | 10,182,502 | 1.54 | 56,892 | 10,393,575 | 2.21 | 56,631 | 10,371,930 | 2.21 | 54,115 | 9,231,808 | 1.45 | 53,944 | 9,167,433 | 1.45 |
| **Class I - LINE** | **CRE-Odin** | 94 | 14,064 | 0.00 | 84 | 15,312 | 0.00 | 75 | 11,722 | 0.00 | 90 | 17,300 | 0.00 | 83 | 13,373 | 0.00 | 86 | 13,980 | 0.00 | 65 | 10,184 | 0.00 | 59 | 9,659 | 0.00 |
|  | **L1** | 14,694 | 7,500,224 | 1.13 | 14,831 | 7,449,521 | 1.10 | 14,643 | 7,400,586 | 1.15 | 14,818 | 7,431,521 | 1.12 | 14,807 | 7,371,346 | 1.57 | 14,818 | 7,269,016 | 1.55 | 13,766 | 6,800,834 | 1.07 | 13,839 | 6,727,256 | 1.06 |
|  | **L1-Tx1** | 686 | 39,433 | 0.01 | 705 | 40,762 | 0.01 | 707 | 36,844 | 0.01 | 710 | 41,509 | 0.01 | 201 | 16,837 | 0.00 | 201 | 16,791 | 0.00 | 713 | 37,819 | 0.01 | 713 | 36,360 | 0.01 |
|  | **L2** | 4,449 | 912,948 | 0.14 | 4,363 | 909,404 | 0.13 | 4,492 | 940,263 | 0.15 | 4,374 | 873,203 | 0.13 | 3,580 | 608,119 | 0.13 | 3,535 | 612,207 | 0.13 | 4,837 | 869,259 | 0.14 | 4,791 | 868,574 | 0.14 |
|  | **Penelope** | 111 | 27,753 | 0.00 | 112 | 28,118 | 0.00 | 114 | 26,949 | 0.00 | 107 | 27,010 | 0.00 | 87 | 15,364 | 0.00 | 90 | 18,554 | 0.00 | 104 | 22,853 | 0.00 | 101 | 20,827 | 0.00 |
|  | **R2** | 21 | 3,093 | 0.00 | 21 | 3,254 | 0.00 | 22 | 4,011 | 0.00 | 19 | 3,156 | 0.00 | 21 | 3,392 | 0.00 | 19 | 3,271 | 0.00 | 21 | 2,351 | 0.00 | 24 | 3,212 | 0.00 |
|  | **RTE-BovB** | 5,128 | 1,990,055 | 0.30 | 5,088 | 1,990,900 | 0.30 | 5,109 | 2,004,777 | 0.31 | 5,178 | 2,041,851 | 0.31 | 4,868 | 971,630 | 0.21 | 4,901 | 979,992 | 0.21 | 4,622 | 1,618,409 | 0.25 | 4,608 | 1,611,596 | 0.26 |
|  | **Rex-Babar** | 244 | 41,887 | 0.01 | 248 | 42,139 | 0.01 | 248 | 42,062 | 0.01 | 252 | 42,982 | 0.01 | 232 | 39,392 | 0.01 | 230 | 38,128 | 0.01 | 252 | 45,056 | 0.01 | 252 | 43,254 | 0.01 |
| **Class I - LTR** | **Cassandra** | 14,541 | 3,567,651 | 0.54 | 14,680 | 3,578,420 | 0.53 | 14,265 | 3,475,657 | 0.54 | 14,578 | 3,553,212 | 0.54 | 14,545 | 3,559,215 | 0.76 | 14,491 | 3,554,224 | 0.76 | 11,863 | 2,832,447 | 0.44 | 11,818 | 2,823,980 | 0.45 |
|  | **Caulimovirus** | 2,432 | 2,296,786 | 0.34 | 2,371 | 2,243,598 | 0.33 | 2,318 | 2,162,013 | 0.34 | 2,322 | 2,192,676 | 0.33 | 1,992 | 1,543,416 | 0.33 | 1,936 | 1,537,459 | 0.33 | 2,910 | 3,005,144 | 0.47 | 2,942 | 3,018,072 | 0.48 |
|  | **Copia** | 97,250 | 98,675,978 | 14.81 | 98,030 | 100,860,386 | 14.95 | 94,464 | 94,247,433 | 14.64 | 96,298 | 96,910,244 | 14.66 | 80,905 | 26,242,317 | 5.58 | 80,529 | 26,269,087 | 5.60 | 103,157 | 98,499,741 | 15.45 | 102,137 | 97,399,722 | 15.41 |
|  | **DIRS** | 120 | 19,547 | 0.00 | 117 | 18,992 | 0.00 | 118 | 19,000 | 0.00 | 112 | 18,120 | 0.00 | 112 | 17,361 | 0.00 | 116 | 18,276 | 0.00 | 103 | 16,348 | 0.00 | 105 | 17,029 | 0.00 |
|  | **ERV1** | 184 | 14,488 | 0.00 | 170 | 13,046 | 0.00 | 188 | 19,050 | 0.00 | 187 | 18,197 | 0.00 | 182 | 18,045 | 0.00 | 185 | 18,320 | 0.00 | 192 | 17,755 | 0.00 | 194 | 18,424 | 0.00 |
|  | **ERV4** | 78 | 10,974 | 0.00 | 74 | 10,584 | 0.00 | 75 | 10,057 | 0.00 | 70 | 9,709 | 0.00 | 70 | 9,920 | 0.00 | 67 | 10,371 | 0.00 | 55 | 7,195 | 0.00 | 49 | 5,659 | 0.00 |
|  | **ERVK** | 76 | 10,243 | 0.00 | 74 | 9,579 | 0.00 | 82 | 11,315 | 0.00 | 80 | 10,229 | 0.00 | 79 | 10,304 | 0.00 | 79 | 9,735 | 0.00 | 88 | 11,070 | 0.00 | 82 | 10,530 | 0.00 |
|  | **Gypsy** | 292,751 | 180,524,618 | 27.09 | 300,035 | 185,268,735 | 27.45 | 272,371 | 168,443,853 | 26.16 | 285,886 | 176,920,149 | 26.76 | 199,171 | 73,619,551 | 15.65 | 198,397 | 73,053,739 | 15.58 | 249,684 | 162,869,267 | 25.55 | 244,840 | 159,981,019 | 25.32 |
|  | **Pao** | 482 | 211,152 | 0.03 | 483 | 202,479 | 0.03 | 479 | 200,105 | 0.03 | 488 | 215,719 | 0.03 | 499 | 197,757 | 0.04 | 503 | 206,188 | 0.04 | 475 | 195,023 | 0.03 | 477 | 194,082 | 0.03 |
|  | **Unknown** | 19,815 | 4,301,019 | 0.65 | 19,728 | 4,259,505 | 0.63 | 19,257 | 4,146,711 | 0.64 | 19,545 | 4,196,446 | 0.63 | 18,421 | 3,890,097 | 0.83 | 18,303 | 3,851,515 | 0.82 | 19,665 | 4,285,149 | 0.67 | 19,572 | 4,290,227 | 0.68 |
| **Class I - SINE** | **B2** | 385 | 24,056 | 0.00 | 379 | 22,995 | 0.00 | 388 | 23,968 | 0.00 | 406 | 25,426 | 0.00 | 400 | 25,195 | 0.01 | 414 | 26,326 | 0.01 | 425 | 27,072 | 0.00 | 425 | 26,651 | 0.00 |
|  | **L1** | 1,089 | 412,661 | 0.06 | 1,069 | 407,565 | 0.06 | 1,061 | 405,625 | 0.06 | 1,093 | 416,247 | 0.06 | 1,113 | 426,548 | 0.09 | 1,118 | 432,090 | 0.09 | 1,106 | 409,139 | 0.06 | 1,100 | 409,337 | 0.06 |
|  | **MIR** | 62 | 5,081 | 0.00 | 61 | 5,165 | 0.00 | 64 | 5,458 | 0.00 | 64 | 5,498 | 0.00 | 62 | 5,048 | 0.00 | 67 | 5,244 | 0.00 | 65 | 5,171 | 0.00 | 57 | 4,625 | 0.00 |
|  | **tRNA-RTE** | 1,724 | 236,936 | 0.04 | 1,708 | 233,231 | 0.03 | 1,689 | 230,802 | 0.04 | 1,725 | 236,685 | 0.04 | 1,600 | 217,738 | 0.05 | 1,606 | 217,965 | 0.05 | 1,051 | 129,803 | 0.02 | 1,064 | 130,577 | 0.02 |
|  | **Unknown** | 497 | 59,968 | 0.01 | 490 | 59,109 | 0.01 | 480 | 60,062 | 0.01 | 492 | 59,323 | 0.01 | 501 | 60,304 | 0.01 | 494 | 59,529 | 0.01 | 510 | 60,447 | 0.01 | 506 | 60,515 | 0.01 |
| **Satellite DNA** | | 1,431 | 369,345 | 0.06 | 1,130 | 263,663 | 0.04 | 1,265 | 256,905 | 0.04 | 1,383 | 286,593 | 0.04 | 2,046 | 438,923 | 0.09 | 2,535 | 551,610 | 0.12 | 1,236 | 281,182 | 0.04 | 1,481 | 339,337 | 0.05 |
| **Simple repeat** | | 1,841 | 334,819 | 0.05 | 1,859 | 329,416 | 0.05 | 1,775 | 322,835 | 0.05 | 1,784 | 325,027 | 0.05 | 1,806 | 325,308 | 0.07 | 1,879 | 335,196 | 0.07 | 1,901 | 330,264 | 0.05 | 1,925 | 343,083 | 0.05 |
| **Unknown** | | 228,718 | 35,846,057 | 5.38 | 230,066 | 36,078,307 | 5.35 | 224,234 | 35,253,021 | 5.47 | 228,457 | 35,836,145 | 5.42 | 218,999 | 34,879,730 | 7.42 | 219,073 | 35,091,803 | 7.48 | 224,613 | 35,536,221 | 5.57 | 223,811 | 35,500,925 | 5.62 |
| **Total repeats** | | 995,461 | 413,187,905 | 62.01 | 1,004,232 | 419,953,333 | 62.23 | 962,773 | 394,482,899 | 61.26 | 987,906 | 407,582,050 | 61.65 | 859,103 | 220,154,920 | 46.81 | 857,848 | 219,609,159 | 46.84 | 951,724 | 391,667,568 | 61.44 | 944,370 | 387,176,187 | 61.27 |

**Table S2**: N repeats summary of *Malus coronaria* (PI590014) and *M. ioensis* (PI590015) haplomes, and of previously-assembled *M. angustifolia* and *M. fusca* haplomes [3,5].

| **Genome** | **Assembly size** | **N gap total length** |
| --- | --- | --- |
| coronaria haploid 1 | 666,594,626 | 304,500 |
| coronaria haploid 2 | 675,532,332 | 295,000 |
| ioensis haploid 1 | 644,188,171 | 288,500 |
| ioensis haploid 2 | 661,368,478 | 294,500 |
| Malus angustifolia haploid 1 | 705,089,958 | 234,793,148 |
| Malus angustifolia haploid 2 | 703,144,148 | 234,311,768 |
| Malus fusca haploid 1 | 637,459,684 | 3,200 |
| Malus fusca haploid 2 | 631,922,950 | 2,800 |

**Table S3:** Gene annotation statistics of *Malus coronaria* (PI590014) and *M. ioensis* (PI590015) haplomes, and of previously-assembled *M. angustifolia* and *M. fusca* haplomes [3,5].

| **BUSCO estimate** | **coronaria haploid 1** | | **coronaria haploid 2** | | **coronaria phased diploid** | | **ioensis haploid 1** | | **ioensis haploid 2** | | **ioensis phased diploid** | | **Malus angustifolia haploid 1** | | **Malus angustifolia haploid 2** | | **Malus angustifolia phased diploid** | | **Malus fusca haploid 1** | | **Malus fusca haploid 2** | | **Malus fusca phased diploid** | |
| --- | --- | --- | --- | --- | --- | --- | --- | --- | --- | --- | --- | --- | --- | --- | --- | --- | --- | --- | --- | --- | --- | --- | --- | --- |
| **Data set** | **genome** | **genes** | **genome** | **genes** | **genome** | **genes** | **genome** | **genes** | **genome** | **genes** | **genome** | **genes** | **genome** | **genes** | **genome** | **genes** | **genome** | **genes** | **genome** | **genes** | **genome** | **genes** | **genome** | **genes** |
| **All complete %** | 97.77 | 96.59 | 97.71 | 96.90 | 98.39 | 98.02 | 97.15 | 95.79 | 97.89 | 97.09 | 98.51 | 97.89 | 98.70 | 97.71 | 98.57 | 97.77 | 98.95 | 98.45 | 98.70 | 98.02 | 98.82 | 98.33 | 98.82 | 98.64 |
| **Single complete %** | 62.45 | 65.18 | 62.76 | 65.30 | 2.48 | 3.47 | 61.96 | 64.75 | 62.14 | 64.31 | 3.22 | 3.66 | 62.02 | 64.75 | 62.14 | 64.56 | 1.55 | 2.54 | 61.65 | 63.14 | 61.52 | 63.26 | 0.81 | 1.92 |
| **Multiple complete %** | 35.32 | 31.42 | 34.95 | 31.60 | 95.91 | 94.54 | 35.19 | 31.05 | 35.75 | 32.77 | 95.29 | 94.23 | 36.68 | 32.97 | 36.44 | 33.21 | 97.40 | 95.91 | 37.05 | 34.88 | 37.30 | 35.07 | 98.01 | 96.72 |
| **Fragmented %** | 1.05 | 0.93 | 0.99 | 0.74 | 0.81 | 0.43 | 1.12 | 0.99 | 0.87 | 0.37 | 0.81 | 0.37 | 0.56 | 0.56 | 0.62 | 0.25 | 0.56 | 0.37 | 0.68 | 0.56 | 0.68 | 0.50 | 0.68 | 0.43 |
| **Missing %** | 1.18 | 2.48 | 1.30 | 2.35 | 0.81 | 1.55 | 1.73 | 3.22 | 1.24 | 2.54 | 0.68 | 1.73 | 0.74 | 1.73 | 0.81 | 1.98 | 0.50 | 1.18 | 0.62 | 1.43 | 0.50 | 1.18 | 0.50 | 0.93 |
| **BUSCO gene number** | 1614 | 1614 | 1614 | 1614 | 1614 | 1614 | 1614 | 1614 | 1614 | 1614 | 1614 | 1614 | 1614 | 1614 | 1614 | 1614 | 1614 | 1614 | 1614 | 1614 | 1614 | 1614 | 1614 | 1614 |
| **All complete** | 1578 | 1559 | 1577 | 1564 | 1588 | 1582 | 1568 | 1546 | 1580 | 1567 | 1590 | 1580 | 1593 | 1577 | 1591 | 1578 | 1597 | 1589 | 1593 | 1582 | 1595 | 1587 | 1595 | 1592 |
| **Single complete** | 1008 | 1052 | 1013 | 1054 | 40 | 56 | 1000 | 1045 | 1003 | 1038 | 52 | 59 | 1001 | 1045 | 1003 | 1042 | 25 | 41 | 995 | 1019 | 993 | 1021 | 13 | 31 |
| **Multiple complete** | 570 | 507 | 564 | 510 | 1548 | 1526 | 568 | 501 | 577 | 529 | 1538 | 1521 | 592 | 532 | 588 | 536 | 1572 | 1548 | 598 | 563 | 602 | 566 | 1582 | 1561 |
| **Fragmented** | 17 | 15 | 16 | 12 | 13 | 7 | 18 | 16 | 14 | 6 | 13 | 6 | 9 | 9 | 10 | 4 | 9 | 6 | 11 | 9 | 11 | 8 | 11 | 7 |
| **Missing** | 19 | 40 | 21 | 38 | 13 | 25 | 28 | 52 | 20 | 41 | 11 | 28 | 12 | 28 | 13 | 32 | 8 | 19 | 10 | 23 | 8 | 19 | 8 | 15 |

**Table S4:** Summary statistics of the identified structural variation in *Malus coronaria* PI590014, *M. ioensis* PI590015, and of previously-assembled *M. fusca* haplomes 1 and 2 [5].

| species | variation type | size interval (bp) | count | total size (kbp) | average size (kbp) |
| --- | --- | --- | --- | --- | --- |
| Coronaria | Inversions | 50 | 0 | 0.0 | 0.0 |
| Coronaria | Inversions | 100 | 87 | 48.1 | 0.6 |
| Coronaria | Inversions | 1,000 | 72 | 292.9 | 4.1 |
| Coronaria | Inversions | 10,000 | 182 | 85,462.2 | 469.6 |
| Coronaria | Translocations | 50 | 0 | 0.0 | 0.0 |
| Coronaria | Translocations | 100 | 1,776 | 1,125.0 | 0.6 |
| Coronaria | Translocations | 1,000 | 2,670 | 8,279.4 | 3.1 |
| Coronaria | Translocations | 10,000 | 153 | 3,514.0 | 23.0 |
| Coronaria | Insertions | 50 | 5,607 | 380.6 | 0.1 |
| Coronaria | Insertions | 100 | 4,420 | 889.7 | 0.2 |
| Coronaria | Insertions | 1,000 | 71 | 280.9 | 4.0 |
| Coronaria | Insertions | 10,000 | 5 | 137.8 | 27.6 |
| Coronaria | Deletions | 50 | 5,514 | 376.2 | 0.1 |
| Coronaria | Deletions | 100 | 4,610 | 906.1 | 0.2 |
| Coronaria | Deletions | 1,000 | 84 | 345.2 | 4.1 |
| Coronaria | Deletions | 10,000 | 9 | 226.0 | 25.1 |
| Ioensis | Inversions | 50 | 0 | 0.0 | 0.0 |
| Ioensis | Inversions | 100 | 82 | 44.2 | 0.5 |
| Ioensis | Inversions | 1,000 | 71 | 268.2 | 3.8 |
| Ioensis | Inversions | 10,000 | 176 | 87,007.5 | 494.4 |
| Ioensis | Translocations | 50 | 0 | 0.0 | 0.0 |
| Ioensis | Translocations | 100 | 1,682 | 1,077.6 | 0.6 |
| Ioensis | Translocations | 1,000 | 2,591 | 8,034.5 | 3.1 |
| Ioensis | Translocations | 10,000 | 146 | 3,431.8 | 23.5 |
| Ioensis | Insertions | 50 | 5,563 | 378.7 | 0.1 |
| Ioensis | Insertions | 100 | 4,318 | 863.9 | 0.2 |
| Ioensis | Insertions | 1,000 | 77 | 306.8 | 4.0 |
| Ioensis | Insertions | 10,000 | 12 | 210.4 | 17.5 |
| Ioensis | Deletions | 50 | 5,502 | 375.2 | 0.1 |
| Ioensis | Deletions | 100 | 4,622 | 901.8 | 0.2 |
| Ioensis | Deletions | 1,000 | 76 | 299.4 | 3.9 |
| Ioensis | Deletions | 10,000 | 8 | 126.7 | 15.8 |
| Fusca | Inversions | 50 | 0 | 0.0 | 0.0 |
| Fusca | Inversions | 100 | 134 | 69.9 | 0.5 |
| Fusca | Inversions | 1,000 | 93 | 272.7 | 2.9 |
| Fusca | Inversions | 10,000 | 146 | 65,911.2 | 451.4 |
| Fusca | Translocations | 50 | 0 | 0.0 | 0.0 |
| Fusca | Translocations | 100 | 1,414 | 908.5 | 0.6 |
| Fusca | Translocations | 1,000 | 2,444 | 7,938.5 | 3.2 |
| Fusca | Translocations | 10,000 | 147 | 3,629.5 | 24.7 |
| Fusca | Insertions | 50 | 6,254 | 425.2 | 0.1 |
| Fusca | Insertions | 100 | 6,204 | 1,474.8 | 0.2 |
| Fusca | Insertions | 1,000 | 154 | 473.3 | 3.1 |
| Fusca | Insertions | 10,000 | 15 | 227.8 | 15.2 |
| Fusca | Deletions | 50 | 6,622 | 450.8 | 0.1 |
| Fusca | Deletions | 100 | 7,080 | 1,629.7 | 0.2 |
| Fusca | Deletions | 1,000 | 181 | 581.9 | 3.2 |
| Fusca | Deletions | 10,000 | 15 | 949.8 | 63.3 |
